# Complete mitochondrial genome of *Bosmina fatalis* (Cladocera: Bosminidae) and its phylogenetic analysis

**DOI:** 10.1080/23802359.2021.1959442

**Published:** 2021-08-05

**Authors:** Wenzhi Wei, Kai Zhang, Qun Shi

**Affiliations:** College of Animal Science and Technology, Yangzhou University, Yangzhou, China

**Keywords:** *Bosmina fatalis*, complete mitochondrial DNA, phylogenetic relationship

## Abstract

*Bosmina* is a globally distributed zooplankton that adapts to a eutrophic environment. In this study, we cultivated monoclonal *Bosmina fatalis* and determined its complete mitochondrial gene sequence using the Illumina HiSeq 4000 platform. It was 15,209 bp in length, including 13 protein-coding genes, 22 tRNA genes, and 2 rRNA genes, with the A + T content (69.2%) significantly higher than the G + C content (30.9%). All 22 typical tRNA genes had a classical cloverleaf structure, except for tRNAIle. The complete mitochondrial genome of nine other cladoceran species was used to reconstruct a phylogenetic tree, showing that *B. fatalis* has a closer relationship with *Daphnia* than other cladocerans.

*Bosmina* is a filter-feeding zooplankton distributed worldwide (Goulden and Frey 1963). In freshwater ecosystems, *Bosmina* plays an important role because it can feed on bacteria, phytoplankton, and organic debris and it is also the main food source for fish and other invertebrates (DeMott 1982; Adamczuk 2012). In addition, *Bosmina* has a hard shell, which can be well protected in the bottom mud of the lake. It is an ideal material for studying ancient limnology and genetics (Taylor et al. 2002). At present, only a small number of mitochondrial DNA sequences of *B. fatalis* are available in GenBank, such as COI and 16S. In this study, the complete mitochondrial gene sequence of *B. fatalis* has been reported, which provides baseline information for phylogenetic status of *B. fatalis* in cladocerans.

*Bosmina fatalis* was collected from Gaoyou Lake (N32°42′, E119°25′) (Wang et al. 2019), cultivated monoclonal in the laboratory, and thousands of individuals were collected, and preserved at −80 °C. Total genomic DNA was extracted using TRIzol™ Reagent (Invitrogen Corporation, Carlsbad, CA), and next-generation sequencing was performed using the Illumina HiSeq 4000 platform (2 × 150 bp) at Shanghai Biozeron Biotech. Co., Ltd. (Shanghai, China). Raw reads were filtered by Trimmomatic 0.39 and assembled using SPAdes v3.10.1 and GapCloser v1.12 with available invertebratemitochondrial genomes as references. The MITOS server was used to predict the mitochondrial genome. The protein-coding gene (PCG) was annotated by blastp analysis with the protein database (NR, Swiss-Prot, eggNOG, KEGG, and GO). The complete mitochondrial genome of *B. fatalis* and nine other cladoceran species were used to construct a phylogenetic tree with the maximum-likelihood (ML) method and the Kimura2-parameter model based on 13 PCGs implemented in the MEGA version 7.0 (Kumar et al. 2016).

The total length of the mitochondrial genome was 15,209 bp (GenBank accession no. MW770308), including 13 PCGs, 22 tRNA genes, and2 rRNA genes (16S rRNA and 12S rRNA). Among them, 23 genes were encoded by the J chain, and the other 14 genes were encoded by the N chain. The mitochondrial genome of *B. fatalis* contains A (32.2%), G (15.2%), T (37.0%), and C (15.7%) with A + T biased composition.

All 22 tRNA genes ranged from 63 bp to 72 bp in size, except for tRNAIle (36 bp), showing the typical cloverleaf secondary structures except for tRNAIle, the 16 srRNA and 12 srRNA which were separated by tRNAVal, were 1381 bp and 755 bp in length, respectively.

The placement of *B. fatalis* among 10 cladoceran species with complete mitochondrial genomes is shown in Figure 1. Among these 10 cladoceran species, *B. fatalis* has a closer relationship with *Daphnia* than other cladocerans.

**Figure 1.**
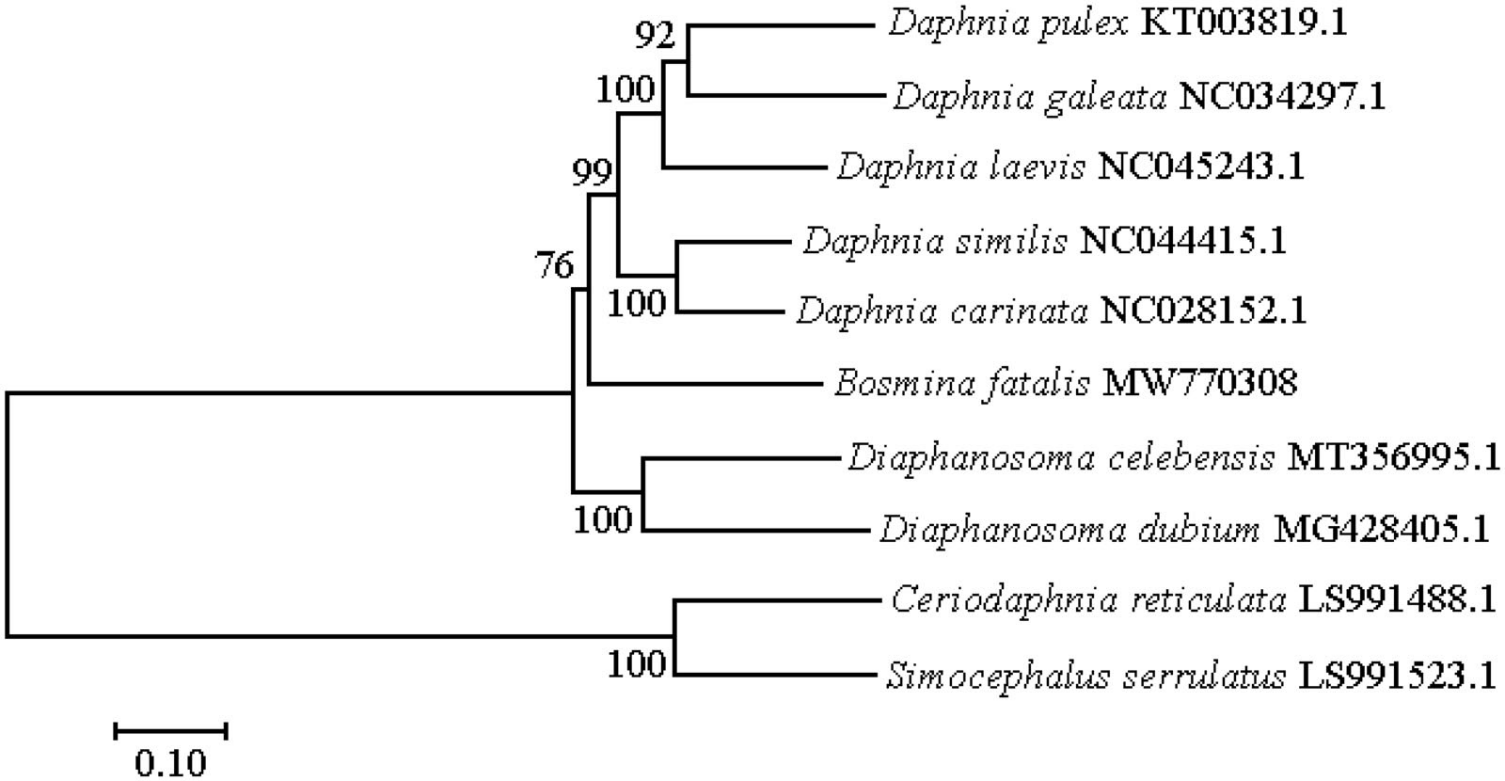
Phylogenetic tree based on complete mitochondrial genome of the *B. fatalis* and 9 other cladoceran species by the maximum-likelihood (ML) method.

## Data Availability

The data that support the findings of this study are available in the NCBI database at https://www.ncbi.nlm.nih.gov, reference number [MW770308].
